# A feasibility assessment of a traumatic brain injury predictive modelling tool at Kilimanjaro Christian Medical Center and Duke University Hospital

**DOI:** 10.1371/journal.pgph.0002154

**Published:** 2023-11-28

**Authors:** Paige O’Leary, Alexis Domeracki, Julius Raymond, Arthi Kozhumam, Victoria Macha, Francis Sakita, Valerie Krym, Joao Riccardo Nickenig Vissoci, Catherine Staton

**Affiliations:** 1 Department of Emergency Medicine, Duke University School of Medicine, Durham, North Carolina, United States of America; 2 Duke University School of Medicine, Durham, North Carolina, United States of America; 3 Kilimanjaro Christian Medical Center, Moshi, Tanzania; 4 Northwestern University Feinburg School of Medicine, Chicago, Illinois, United States of America; 5 Department of Emergency Medicine, University of Toronto Medical School, Toronto, Ontario, Canada; 6 Duke Global Health Institute, Duke University, Durham, North Carolina, United States of America; Royal Infirmary of Edinburgh and University of Edinburgh, UNITED KINGDOM

## Abstract

Traumatic brain injury (TBI) is the most common cause of death and disability globally. TBI, which disproportionately affects low middle-income countries (LMIC), uses significant amounts of health system resources in costly care and management. Innovative solutions are required to address this high burden of TBI. One possible solution is prognostic models which enhance diagnostic ability of physicians, thereby helping to tailor treatments more effectively. This study aims to evaluate the feasibility of a TBI prognostic model developed in Tanzania for use by Kilimanjaro Christian Medical Center (KCMC) healthcare providers and Duke-affiliated healthcare providers using human centered design methodology. Duke participants were included to gain insight from a different context with more established practices to inform the TBI tool implementation strategy at KCMC. To evaluate the feasibility of integrating the TBI tool into potential workflows, co-design interviews were conducted with emergency physicians and nursing staff at KCMC and Duke. Qualitatively, the TBI tool was assessed using human centered design (HCD) techniques. Our research design methods were created using the Consolidated Framework for Implementation Research which considers overarching characteristics of successful implementation to contribute to theory development and verification of implementation strategies across multiple contexts. Our knowledge translation method was guided using the knowledge-to-action framework. Of the 21 participants interviewed, 12 were associated with Duke Hospital, and 9 from Kilimanjaro Christian Medical Centre. Emerging from the data were 6 themes that impacted the implementation of the TBI tool: access, barriers, facilitators, use of the TBI tool, outer setting, and inner setting. To our knowledge, this is the first study to investigate the pre-implementation of a sub-Saharan Africa (SSA) data- based TBI prediction tool using human centered design methodology. Findings of this study will aid in determining under what conditions a TBI prognostic model intervention will work at KCMC.

## Introduction

Traumatic brain injury (TBI) is the leading cause of morbidity and disability globally [1–3]. Low middle-income countries (LMICs) experience three times as many TBIs compared to high income countries (HICs) [4]. Within LMICs, one of the highest rates of TBIs occurs in sub-Saharan Africa(SSA) and the incidence of TBI is projected to increase significantly by 2050 [5]. In 2017, the rate of mortality among severe TBI patients in Moshi, Tanzania was 47%, which was significantly higher compared to TBI mortality rates in HICs [6]. Limited resources and a lack of neurosurgical capacity in Tanzania mean innovative solutions, such as decision tools, are required to address this high burden of TBI. Combatting growing rates of TBI and poor TBI outcomes is therefore an increasingly important public health concern.

The burden of TBI in the United States (US), a HIC, affects 1.7 million individuals per year, contributes to 1-in-3 injury-related deaths, and disproportionately affects indigenous and black communities [7]. However, in HICs patient outcomes are substantially better and the use of decision tools is more established compared to LMICs. Therefore, HICs could 1) offer insight into making innovative solutions appropriate for settings that are developing workflows and patient processes and 2) improve their use of decision tools, including for TBI patient outcomes.

The standard of care for TBI patients varies as there are a multitude of factors that impact patient management. Prognostic models, a type of decision tool, could provide a solution to standardizing patient care under conditions of limited capacity, since the models enhance diagnostic ability of physicians, thereby helping to tailor treatments more effectively [8]. Currently, few physicians are confident in their ability to predict TBI patient outcomes. In a survey of doctors from HICs and LMICs, only 37% were confident in their ability to assess prognosis among head injury patients [9]. Prognostic models have been proven capable of predicting patient outcomes with greater accuracy than physicians without decision-support tools [10]. Currently, the quality of prognostic models varies, and many of them lack adequate validation in LMICs [11]. Furthermore, no prognostic models built using patient data from sub-Saharan Africa (SSA) have been implemented in SSA. In 2019, the Global Emergency Medicine Innovation and Implementation Science Research Centre (GEMINI) center developed a TBI prognostic model with a substantial level of accuracy but it remains to be externally validated as a clinical decision support tool, especially in low resource settings [12]. The TBI prognostic model is a web-based application, also referred to as the TBI tool. The TBI tool’s input variables are clinical scores and sociodemographic information, and the outputs are predicted mortality with or without surgical interventions meant to support, but not replace, clinical judgment.

This paper aims to assess the feasibility and practicality of a TBI prognostic model implementation at Kilimanjaro Christian Medical Center (KCMC, Tanzania) and amongst Duke (US) affiliated healthcare providers using human centered design techniques to transfer knowledge between both settings to improve the future implementation of the tool at KCMC. In exploring two separate clinical contexts, this study will facilitate knowledge exchange and identify best practices for implementation of the TBI tool at KCMC.

## Methods

### Study setting and participants

This two-site study took place in Moshi, Tanzania, and North Carolina, USA. KCMC is a tertiary care hospital serving a population of over 15 million people in Moshi, Tanzania. KCMC is the primary point of care for TBI within the Northwestern region [6,13]. The TBI burden is staggering with 1,000 TBI patients annually and nearly one-third of all injury patients present with TBI. In this setting, injury patients are five times more likely to die if they have a TBI [14]. Upon arrival to KCMC suspected TBI patients are evaluated and triaged in the emergency department. The initial assessment of severity and risk of poor outcome is largely based on the healthcare providers skill and intuition [12]. The use of resource-intensive diagnostics (ex. head CT) is limited by cost, which has to be paid prior to delivery of service, as well as the availability and usability of the equipment [6,12]. The second site is Duke University Hospital, a level 1 trauma center, located in North Carolina, USA. In 2021, the hospital admitted 41,000 patients and had 1.3 million outpatients [15]. In North Carolina, there are over 74,000 emergency visits due to TBI per year [16]. Compared to LMICs, TBI patients in HICs have better clinical outcomes and more established clinical procedures.

### Sampling

Purposive sampling and snow-ball sampling were used to recruit participants. Study participants were healthcare providers at KCMC (N = 9), and Duke Hospital (N = 12) involved in the care pathways of TBI patients. Participants included emergency medical doctors, nurses, physician assistants (PA) and medical residents. These subjects are representative of the future end-users of the TBI prognostic model. Participation was voluntary, and all participants provided written consent.

### Study design

This study was a multi-site feasibility study using co-design sessions, an integral part of human centered design (HCD) research. HCD is an emerging research approach that has typically been used in fields outside of health science. HCD holds promise for improving implementation of evidence-based health interventions [17]. The purpose of HCD is to unite end-users with researchers and developers to co-create health products or delivery strategies. The results will then reflect the setting and context of final implementation [18–20]. Participants were asked to reflect on their day-to-day workflow, timelines, and scenarios based on their context specific settings.

### Data collection

Our preliminary work identified two potential situations for TBI tool use: at the acute triaging of the patient, and during the hospital stay in case of any deterioration of clinical status. Two RAs from KCMC and one RA from Duke conducted the co-design sessions at their respective institutions. The RAs did not have any previous relationships to the participants.

Co-design sessions, part of HCD research, were used to seek a deeper insight into the users cultural, emotional, and practical requirements of the TBI tool. The purpose of the co-design sessions was to create an implementation strategy focused on participants, thereby developing interventions catered to future users while facilitating enhanced understanding and ownership of the project. Co-design sessions allowed us to determine if the TBI tool would fit into the day-to day workflow and/or might expedite care at KCMC or Duke Hospital. Human centered design techniques, qualitative methodology and the Consolidation Framework for Implementation Research (CFIR) [21], and knowledge-to-action framework [22] were used to create the individual co-design sessions with healthcare personnel. Qualitative research assistants (RAs), trained by the study investigators, conducted the co-design sessions using WebEx video platform (Duke Hospital) or in private rooms one-on-one (KCMC).

The co-design sessions consisted of three activities: timeline, scenario completion, and rapid fire. Duke Hospital participated in all three sessions with an average of 1.5 hours per session. KCMC participated in timeline and scenario design sessions, excluding rapid fire due to time restrictions from the KCMC Institutional Review Board.

A timeline session asks participants to create a timeline (a visual representation of the flow from one step to the next over a certain period of time) of their typical day at work, starting from when they woke up to when they went to sleep (S1 Fig) [23]. Scenario completion techniques involve participants writing down and contemplating hypothetical scenarios [24]. In this study, participants were asked to write down physical spaces in which they encounter TBI patients. Then they were asked a series of questions for different patient scenarios (mild vs severe TBI) within the physical spaces they had listed (S2 Fig). The final activity, rapid fire, is a compilation of rapid fire and card sorting. Rapid fire involves giving participants time to ‘brain dump’ their thoughts. Card sorting is a common technique in human centered design used to find patterns in how end- users expect to find content or functionality [25]. For the third co-design activity, participants were given three minutes to write down their ideas and then asked to sort their ideas into two piles (S2 Fig).

### Analysis

Interviews were recorded and then transcribed prior to the analysis by RAs. Once interviews were transcribed, the participants’ qualitative responses were structurally coded to identify themes. Codes are words or sentences that represent the content of a specific participant response. The structural codes were developed using the interview guide. To ensure that the codes were appropriate and reproducible, two RAs coded 25% of the Duke Hospital and KCMC transcripts and compared the results. The intercoder reliability score was Kappa = 0.91, meaning that coding by each RA strongly agreed. The themes from the structural codes were identified using the coding reports generated in NVivo12 [26]. Subthemes within the structural codes were identified and collected in a data reduction table (Table 2). Details from the data reduction table were reported as the results.

### Ethics

This study has IRB approval from the Duke Institutional Review Board (Pro00106607) and Kilimanjaro Christian Medical Center Ethics Committee, and NIMR (No. 1229). This study has a minimal level of risk. Informed written consent was obtained from all participants before the co-design sessions. All participants were told that they could drop out of the study at any time without repercussions. Confidentiality of co-design sessions was addressed by using unique identification numbers.

## Results

### Demographics

Of the 21 participants interviewed, 12 were associated with Duke Hospital, and 9 from KCMC (Table 1). All participants included were involved in the care pathways of TBI patients at either hospital. The population at Duke Hospital were mostly white (75%), doctors (50%) with an average of 5.75 (4.4) years of work experience. The Duke population was almost equally male (n = 7) and female (n = 5). At KCMC all participants were black (100%), the majority were nurses (77%) with an average of 5 (3.6) years’ work experience (Table 1). The KCMC population was almost equally male (n = 4) and female (n = 5).

**Table 1:** Participant demographics at Duke Hospital and KCMC.

Variables	KCMC (n = 9)	Duke (n = 12)
Female, N (%)	5 (56.0)	5 (41.7)
Age in years, (SD)	34.8 (11.5)	33.2 (5.4)
Race, N (%)		
White	0 (0)	9 (75)
Asian	0 (0)	2 (16.7)
Black	9 (100)	0 (0)
Mixed Race	0 (0)	1 (8.3)
Positions, N (%)		
Doctors	2 (22.2)	6 (50)
Residents/PAs	0 (0)	3 (25)
Nurses	7 (77.8)	3 (25)
Average years worked, mean (SD)		
Doctors	1 (0)	5.8 (4.4)
Residents/PAs	0 (0)	1.7 (0.9)
Nurses	5 (3.6)	5 (1.6)

### Qualitative findings

Emerging from the data were 6 themes: Access, Barriers, Facilitators, Use of the TBI Tool, Outer Setting, and Inner Setting. Each theme had several subthemes, as described in the below and within Table 2, with exemplar comments found in Table 3.

**Table 2:** Qualitative data themes at Duke and KCMC.

	KCMC	Duke Hospital
Themes	Subthemes	Subthemes
Access	Technology	Technical
	Timing	Non-technical
		Timing for using TBI tool
Barriers	Risks	
	Acceptability	
	Technology in workflow	Technology
		Workflow and current practices
		Utility
Facilitators	Accuracy	
	Workflow	
	Education	Availability
Use of TBI tool	Use of the predictive model outside of the hospital	
	TBI decisions	
	Use of the predictive model in the hospital	
	TBI encounters	Workflow and TBI encounters
Inner Setting	Patients’ perceptions	
	Implementation climate of the hospital	
	Social context of workplace	
		Duke culture
Outer Setting	External policies and incentives	
	Implementation climate of country and region	
	Tanzanian culture	American culture

**Table 3:** Summary of where along the trauma care pathways the TBI tool could be used.

	TBI Severity	Timing with care pathway	Advantages	Disadvantages
KCMC	Mild	Early	On rounds, preparing and receiving ward report, prior to communicating with family	N/A
	Severe	Early		Too time intensive
Duke	Mild	Early, Post-CT	Communicate prognosis with patient	May not be useful
	Severe	Later, intermittently	Tracking progress, updating families, interdepartmental communication	N/A

**Access.** Access to the TBI tool included subthemes of technical access (modality and display preferences), non-technical access (designated champion or hard-copy versions), and timing (when/where in patient care). KCMC participants preferred iPad/tablet relative to phones (low storage) or computers (immobile). Computers were preferred over phones as the TBI tool could be integrated into the charting software used throughout the hospital. A mobile app could allow remote access to patients and providers, but with concerns about storage and payment for mobile data use. Conversely, Duke participants preferred to integrate the TBI tool into the electronic medical record as it saves time, is accessible to EMS, auto-populates some input variables, and could be programmed as an alert for all patients with an acute TBI diagnosis. KCMC participants also suggested including an alert to providers when the TBI tool output suggests prolonged observation.

### KCMC

“*if you developed a tool that can give a probability of this patient losing consciousness … is this much… So*, *if the tool can assist me in saying … maybe I should stay with this patient a little bit longer*.*”*

### Duke

“if it showed up as an alert…where like this patient has a greater than 10 percent chance of needing surgery in the next 24 hours…you need to know right away, and not send the patient home.”

Both Duke and KCMC suggested fewer input variables. Input concerns at KCMC centered on access to computers or tablets, and the equipment to obtain input variables. In general, at KCMC resources are differentially allocated based on TBI severity, potentially resulting in unequal access to the TBI tool. Whereas Duke participants focused more on output preferences with simpler, color-coded output displays to more effectively communicate prognosis with patients and families.

Within the timing subtheme of access, participants thought about where in the care pathway the TBI tool would be used, and if use would differ for mild or severe TBI patients. Table 3 below summarizes participant views. At KCMC, some participants also identified that the most effective time for using the tool was during the first assessment.

“*Oftentimes he (first assessor) biases the whole system so even if a person comes to see the patient later*, *he usually refers and oftentimes tend to be biased by the first person so you go to the diagnosis of the first person*, *very rarely do you change your course because you think the first person might have missed something*.*”*

### Barriers

The co-design activities proactively identify barriers to implementation at Duke Hospital such as programming of the tool, workflow, acceptability, utility, and risks. Whereas at KCMC, the co-design activities predicted that barriers to implementing the TBI tool were three-fold: technology in the workflow, acceptability, and risks.

KCMC participants considered the number of inputs and cost/access to the internet to be barriers to implementation of the TBI tool. Additional barriers regarding acceptability of the tool were increased workload and the following sociobehavioral barriers: the questioning of a provider’s knowledge, feeling like the tool is a waste of time, and general difficulty with behavioral change.

“*The biggest barrier that I can see is the tool demands a lot from the doctor*, *it wants you to do things the proper way and we often don’t do things the way they should be done*. *Why*? *Because we think we are very experienced*, *we think that doing it will eat away our time and then we are creatures of habits…the issue of the tool being some sort of an extra burden to a doctor … how you can make the doctor see the tool as a friend*.*”*

Similarly, Duke participants foresaw the number of inputs and internet access as potential barriers but added that the tool was devised using KCMC data which may not be accurate for the Duke patient population. Participants also commented on workflow and poor implementation of new technology in general, as well as lack of universal implementation across hospital departments. Emergency providers considered surgical prognosis beyond their immediate scope and therefore would be less likely to use the tool.

### Facilitators

Accuracy, workflow, and education were identified by KCMC participants as aspects of the tool and the workplace that would facilitate its implementation. At Duke, the identified facilitators were accuracy, workflow, and availability.

At KCMC, accuracy was important for proper resource allocation. Accuracy in the Duke Hospital context was seen as a requirement for successful implementation and included reliability, utility, clinical significance, and transparency. Participants at Duke desired evidence of improved mortality outcomes and additional outputs relevant to significant clinical outcomes like function after medical intervention.

Duke: “*Will this patient be able to interact or talk afterwards*, *not just death*, *right*? *That would be a big question that I feel like a lot of families would want answered […] if you are able to differentiate between like there’s a 40% chance and a 70% chance*, *that’s going to be a lot more useful of a tool than saying there’s 51% chance and a 52% chance…”*

Duke participants also thought there would be greater implementation success if the tool aligned with the workflow, aligned with the research priorities and if use was mandatory. To align with the current workflow, Duke participants expressed that the TBI tool would need to be integrated into a task that is already being performed, such as the neurological exam. One nurse said “from an assessment standpoint and from a nursing perspective it’s an incredibly easy thing to tack on to what we’re already doing.” Further, participants expressed that decision-making tools are part of their practices already, such as MDCalc, Sepsis watch, PECARN Institute of Health stroke scale, as some examples.

Workflow commentary from the KCMC participants stated that if the tool was able to help navigate patient admission and discharge, as well as patient tracking and trending, it would integrate well into the workflow. KCMC also suggested mandatory use of the TBI tool in charting. Education was also cited as an important facilitator for KCMC, indicating the importance of training and transparency with how the model operates.

### Use: KCMC

Participants shared their experiences with TBI patients and brainstormed how the TBI tool could be used. Specifically, they described TBI encounters, TBI related decisions, and how the TBI tool could be used in their workplace. KCMC participants identified several existing points of difficulty in caring for TBI patients that could be ameliorated by the TBI tool. For example, overcrowding, limited resources, and fewer available providers for patient care and evaluation creates barriers for resource allocation. Lastly, the lack of standard operating procedures for TBI patients makes consistency of care difficult. Participants foresaw the TBI tool as useful for patient management decisions, especially for newly hired staff, and as a tool to facilitate resource allocation. The tool could also trend and summarize patient status, helping to address the challenge of limited time availability with individual patients and prompting providers if unexpected deviation occurs. The TBI tool could also be useful for communicating patient status and prognosis virtually if a provider is not available in the hospital, or between hospital departments.

*“We don’t have a systematic way of saying like when should I supplement with oxygen*, *when should I give inotropic…it is usually dependent on the user so if you are very experienced…then you know that you should supplement oxygen as early as possible… but for the novice or interns who are actually coming in…who also attend these patients they usually not very good at managing them.”*

### Use: Duke

Participants at Duke Hospital shared their experiences with TBI patients to describe TBI encounters, TBI related decisions, and the potential use of predictive modeling tools within and outside of the hospital setting. Within the ED, challenges included diagnosing severity, hectic nature of the ED, unknown baseline status, and triaging based on severity. Additionally, the presence of competing priorities and patient transfer posed challenges throughout the hospital system or during shift change. Whereas strengths included high resources (nursing and surgical providers) and clear protocols for mild and severe TBI treatment.

The range of TBI severity also proposes a range of challenges. Severe TBIs require timely care and intensive nursing resources, especially for impulsive or distracted TBI patients. Mild TBI patients and their loved ones often have difficulty with the lack of empirical evidence supporting their diagnosis. Additionally, determining which mild TBI patients may worsen presents a problem as pointed out by one participant “their symptoms aren’t as obvious, so trying to use a fine-tooth comb to make sure I’m not missing small neuro findings.”

### Inner and outer settings

**Fig 1:**
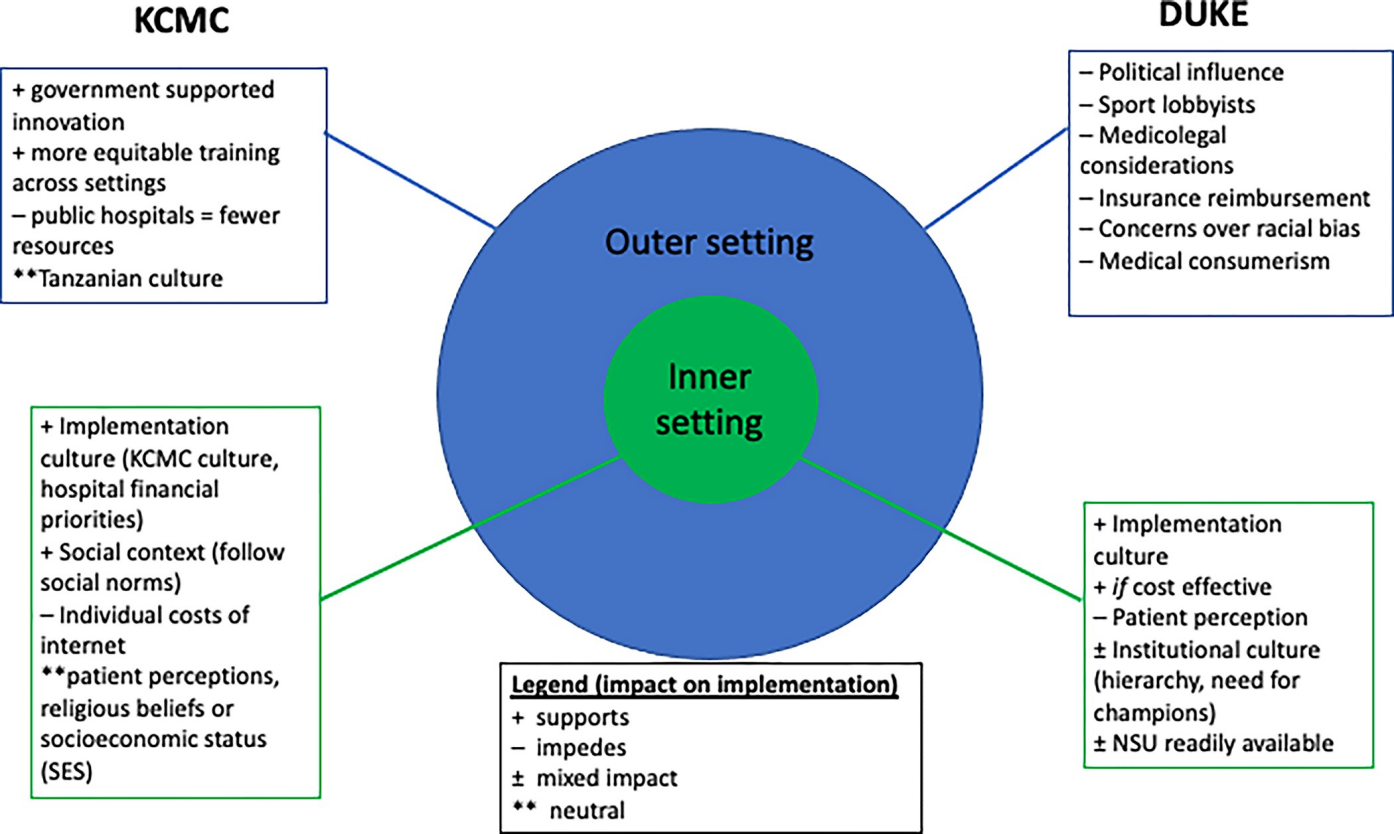
Summary of co-design session findings based on the inner and outer settings defined by the CFIR framework; categorized by the impact of each participant response (supports or impedes implementation).

Participants at both KCMC and Duke were asked to explore the relative impact of the inner setting (workplace culture, patient perception) and the outer setting (national culture, implementation climate of the region, and external incentives) on future implementation of the TBI tool (Fig 1).

The inner setting for Duke culture supports implementation as it aligns with the priorities of research, education, and evidence-based medicine. However, some participants addressed concern over the use of a phone app due to patient perception of phone use for non-medical purposes in the hospital. The potential added value of the TBI tool also supports the implementation climate of the hospital so long as the tool is cost effective and properly sensitized to use resources accordingly.

In co-design sessions at KCMC, the implementation climate of the hospital included KCMC culture, hospital financial priorities, and individual costs. KCMC culture was included in the implementation climate of the hospital, as participants did not report out certain attitudes, tendencies or practices that suggested a strong KCMC specific culture. Instead, participants described an environment that was open to change, which lent more to the subtheme of the hospital’s implementation climate.

The economic priorities of the hospital were unclear with varied responses. Some did not see a connection between the tool and KCMC finances, whereas others thought either existing technology could be used, or new tools would need to be purchased, thereby oppositely impacting financial burden. On an individual basis, participants stated that cost would be a barrier due to phone storage capacity for the app. The majority of participants did not think that the patient perceptions, religious beliefs or socioeconomic status (SES) would impact the implementation of the tool.

In the KCMC outer setting, participants considered the impact of politics, external policies, and incentives to be low for the initial implementation of the TBI tool. Participants focused on two aspects of the Tanzanian healthcare system when considering the implementation climate of their country: training resources and public vs private health centers. As training varies throughout Tanzania, participants thought the TBI tool could help mitigate some of these resource issues that result in untrained personnel.

“… *we have many clinicians or many health care workers who have not had proper training on how to manage patient with TBI so this tool is important in developing countries… it helps so much to guide the clinician or the health care practitioner on what to do and not to miss things*, *so it fills that gap at this point*.*”*

Additionally, the public hospitals were seen to have less accountability and fewer resources, so they might have more issues with implementation. However, participants thought that the TBI tool could help hold public and private healthcare providers accountable. Within Tanzanian culture specifically, participants thought there would be minimal little effect of culture on the implementation of the TBI tool as patients in the hospital are seen as separate from society.

### Similarities and differences between KCMC and Duke Hospital

**Fig 2:**
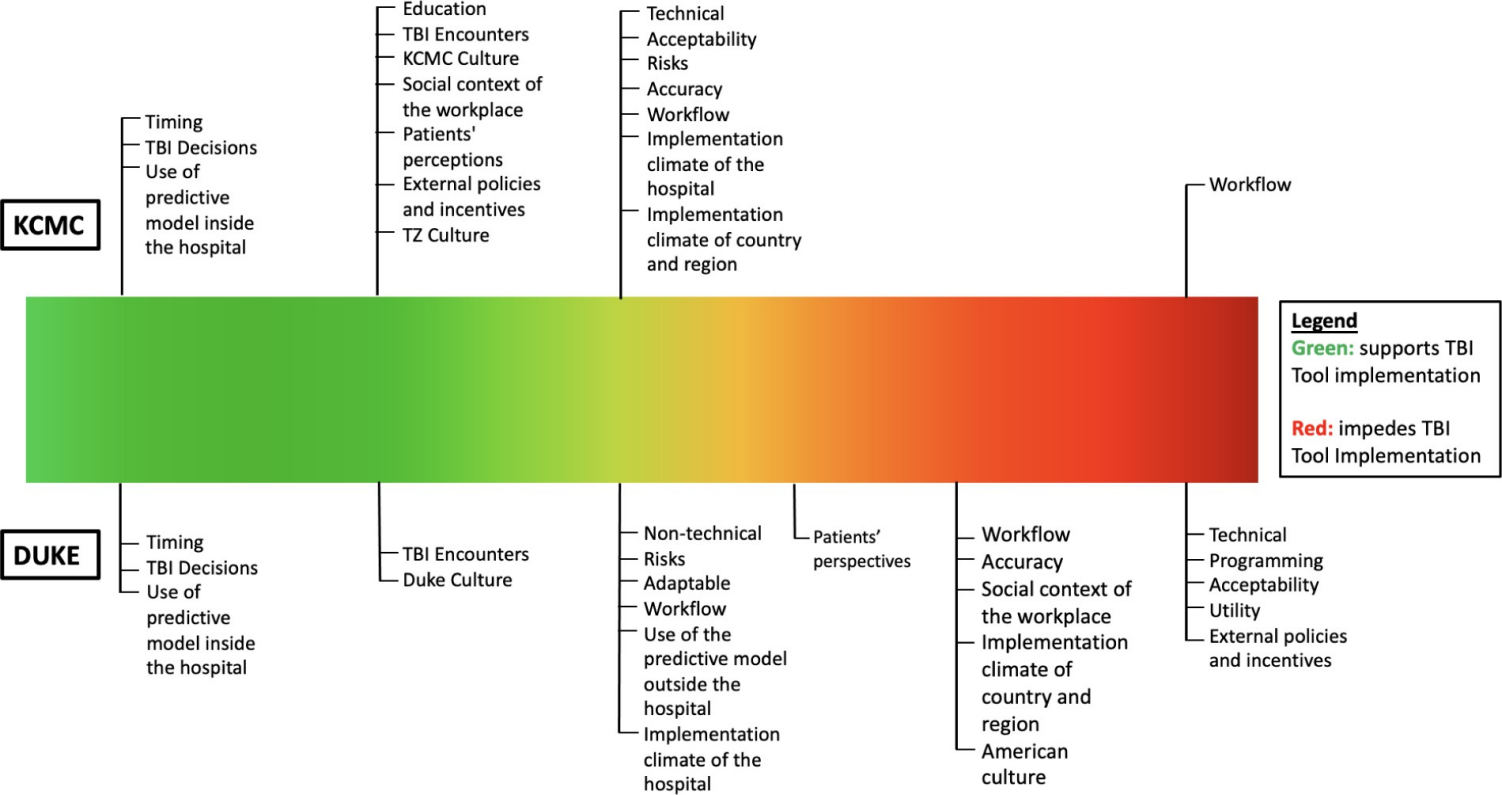
Spectrum of observed impact of subthemes on TBI tool implementation at KCMC and Duke Hospital.

In this study, we compared subthemes between KCMC and Duke Hospital. To visually compare subtheme impact on future implementation, subthemes were ranked on a scale of 1–5 based on codesign sessions with 1 representing barrier to implementation, and 5 representing a facilitator of implementation. Results were then mapped, as seen in Fig 2, to provide a visual spectrum of subtheme impact on implementation. Overall, KCMC had more subthemes that supported the use of the TBI tool as it currently is, compared to Duke Hospital. Lastly, Table 4 summarizes the requirements of changes to the TBI tool that participants thought would yield in positive implementation.

**Table 4:** Summary of the TBI tool requirements participants suggested for the tool to be implemented at KCMC.

Challenge	Solution
Technical	• Reduce number of inputs • Store data • Color display of output
Education	• Workshops • Tool development transparency • Troubleshooting resources
Accuracy	• Education • Experience/ adjusting to using tool
Workflow	• Mandated • Pre-identified champions
Social Context	• Inform other departments. • Hospital wide
Economic properties/cost	• Free • Cost-effectiveness analysis

## Discussion

To our knowledge, this is the first study to investigate the pre-implementation of a SSA data- based TBI prediction tool using human centered design methodology. KCMC participants identified under what conditions the TBI tool could be implemented into their current workflow. Duke Hospital participants provided insights about implementation of the TBI tool which will serve KCMC as their hospital processes continue to advance. Moreover, the co-design findings from the two study sites identified similarities, differences, and learning opportunities to be shared between these two settings as the feasibility study moves into the implementation phase at KCMC. Further, the findings from this study will be used to inform future implementation studies of TBI tools within the Duke health system. Lastly, the lessons learned from comparing the findings between the HIC and LMIC will contribute to an implementation tool kit that utilizes HCD techniques for global health research.

We observed multiple similarities between the responses from participants at Duke and KCMC. The most similar subthemes that impacted implementation of the TBI tool at both study sites included timing, workflow, risks, accuracy, TBI encounters, TBI decisions, in hospital use of the TBI tool, hospital culture, and climate of implementation. Almost all related subthemes supported the use of the TBI tool. However, there were slight differences between the specific information provided per subtheme at KCMC compared to Duke. For example, both sites agreed that their current workflow would support the use of the TBI tool especially if it was mandated, as mandating practices has been shown to improve implementation of hospital-based interventions [27]. They also agreed upon workflow benefit for the following reasons: they see TBI patients, input variables are already collected, and participants could identify potential workflow benefits like hospital admission and patient trends. In contrast, the sites had different rationales to challenge the implementation of the TBI tool. For example, at KCMC the workflow doesn’t consistently use electronic devices. Whereas at Duke employing the TBI tool as a website would impede workflow as they already use EPIC. These findings further support the current literature which highlights the importance of pre-identifying context-specific characteristics of each implementation site [28–30].

Interestingly, the TBI encounters and TBI decisions subthemes had many similarities between study sites, despite the differences between HICs and LMICs. Both sites agreed that the TBI tool could be used for a majority of all TBI patients, though participants had a preference for using the TBI tool with mild or moderate TBIs as severe TBIs typically have a clear care pathway. However, participants said that for patients with expected mortality, the tool could be useful to know when to stop care prior to using exhaustive resources and to provide evidence when they cannot do more for the patient. The TBI tool could overcome the unique challenges that all EDs face when making these decisions to stop care, as typically they lack crucial data concerning the patient’s baseline and future functional outcomes whether they receive intensive care [31]. Other TBI decisions at both sites that would be supported by the TBI tool included resource allocation, trending, long-term prognosis and surgery. The similar TBI decisions highlight that there are challenging decisions in all settings regardless of the resources available, and demonstrates knowledge translation opportunities between HIC and LMIC study sites [32]. Therefore, having a tool to support common decision points would likely be more feasible to implement across different contexts.

Between the two sites, major differences in subtheme impact included technical, acceptability, use of the TBI tool outside the hospital, social context of the workplace, external policies and incentives, and country culture. Most differences *impeded* use of the TBI tool at Duke Hospital and *supported* the use of the TBI tool at KCMC. This disparity suggests that the TBI tool is currently better suited for implementation in the KCMC setting; a promising finding as the tool was developed for a LMIC setting with data from Tanzania. For example, the impact of country culture on the TBI tool use was different between study sites. At Duke hospital the culture of medical consumerism threatened the utility of the TBI tool in the ED, whereas at KCMC the country culture supported the TBI tool. Consumerism-based healthcare as a barrier to implementing decision tools, such as the TBI tool, are relatively new and accompanied by the pressure to satisfy patients. Duke participants expressed that they do not often provide conservative care, and that their patients are their customers that must be satisfied. A similar phenomenon was observed in a study assessing why management of syncope is not value-based across four different hospitals in the US. The study reported that clinicians felt pressure to over test and to provide good customer service, leading to a disregard of the guidelines [33]. This pressure for a consumer approach to medicine is unique to our HIC setting, as there was no reporting of this approach at KCMC. Further, at KCMC, participants viewed the hospital as a silo from society and did not think that the cultural nuances of Tanzania would impact the implementation or use of the TBI tool.

Overall, there was a surprising number of common challenges faced at both study sites. Taken together, participants identified TBI tool characteristics required for implementation. Key challenges to address prior to implementation at KCMC, as summarized in the results, included: technical changes, education, accuracy, workflow integration, accounting for social context, and analyzing the cost effectiveness of using the tool. These challenges have been reported in other mHealth implementation science studies. By identifying these challenges prior to implementation, preliminary adaptations can be made to increase end user buy-in as stakeholders can see their impact on the final TBI tool. Previous studies have shown that participants who helped co-create solutions resulted in designs that scored significantly higher scores for end user benefits and satisfaction with the intervention [17,34,35]; and showed that co-design reduces unintentional reinforcement of inequities [36]. Further, these findings demonstrate how HCD techniques, such as co-design, could potentially overcome some of the challenges of implementation and end-user’s uptake as participants contribute to creating the solution.

There are three main limitations of this study. Firstly, KCMC participants did not do the rapid fire/ pile sort co-design activity due to time restraints. Although we were unable to compare this co-design activity between the two settings, data from the other activities suggested this was not a major limitation as no new subthemes were generated in the rapid fire/pile sort activity among the Duke affiliated participants. Secondly, the composition of the samples was different between the two sites. A majority of the KCMC participants were nurses, whereas the Duke population was more evenly divided across job titles. This difference is appropriate, however, since there are fewer doctors at KCMC compared to Duke Hospital. The samples in each setting reflected the distribution of healthcare providers in that setting. Lastly, at both Duke Hospital and KCMC the sample could be biased, as there was a large time commitment to participate in stage 1, and therefore only people interested in research may have agreed to participate. This is a common bias and we do not know how the characteristics of those who chose not to participate may be different than those who did. However, the newness of the methodology may have mitigated this risk somewhat, as it may have attracted more than the usual research knowledgeable participants.

To our knowledge, this is the first study to investigate the implementation of a SSA data- based tool in SSA, and to compare HCD methodologies between an HIC and LMIC setting in a single study. Currently, the TBI tool is more suitable for KCMC and will be implemented using co-designed solutions presented in this article. KCMC participants identified under what conditions the TBI tool could be implemented into their current workflow. Duke Hospital participants provided insights about implementation of the TBI tool which will serve KCMC as their hospital processes continue to advance. Moreover, the co-design findings from the two study sites allowed for similarities, differences, and learning opportunities to be identified and shared between these two settings as we plan the implementation of the TBI tool at KCMC. Findings from our study will inform the updates made to the TBI tool and implementation strategy for the next stages of the feasibility study at KCMC. Further, these findings can be used to inform future implementation studies of TBI tools within the Duke health system, and health systems abroad.

## Supporting information

S1 Fig Co-design timeline (process mapping) activity. Timeline activity.
(TIF)

S2 Fig Co-design scenario completion and rapid fire (pile sorting) activities. Scenario and rapid fire activities.
(TIF)
