# Supplementary figures and images for: A feasibility assessment of a traumatic brain injury predictive modelling tool at Kilimanjaro Christian Medical Center and Duke University Hospital
Source: PLOS Glob Public Health. 2023 Nov 28;3(11):e0002154. doi: 10.1371/journal.pgph.0002154 (PMC10684081; doi:10.1371/journal.pgph.0002154)

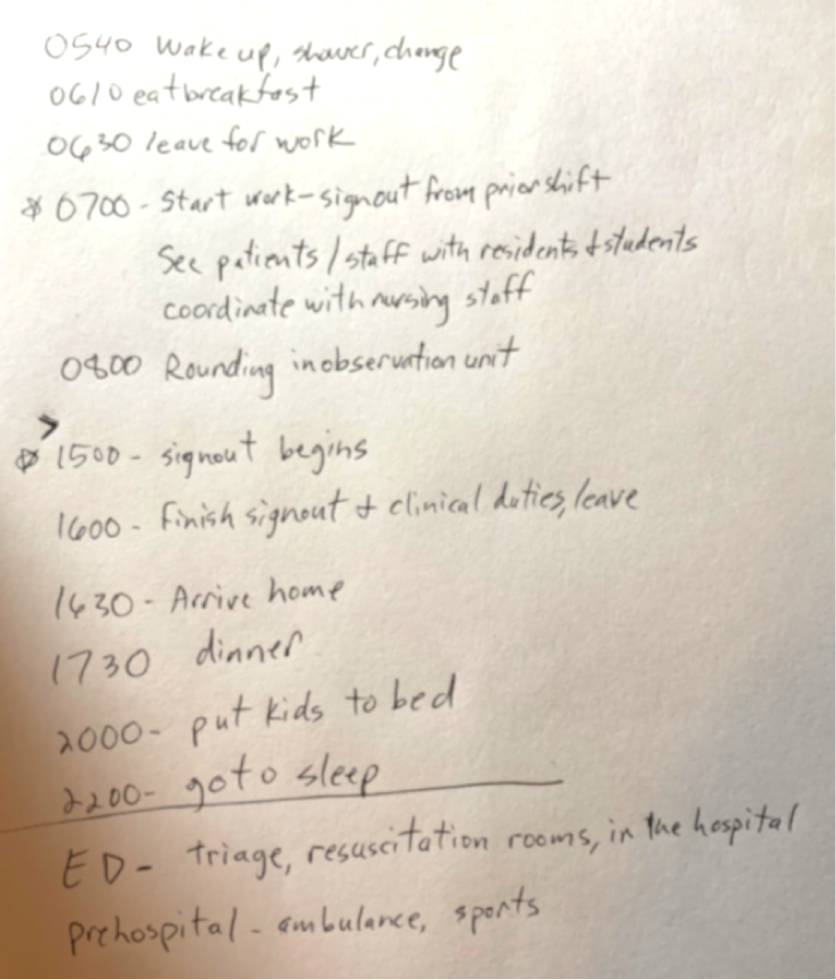

Supplement: S1 Fig — Timeline activity. (TIF) [file pgph.0002154.s001.tif]

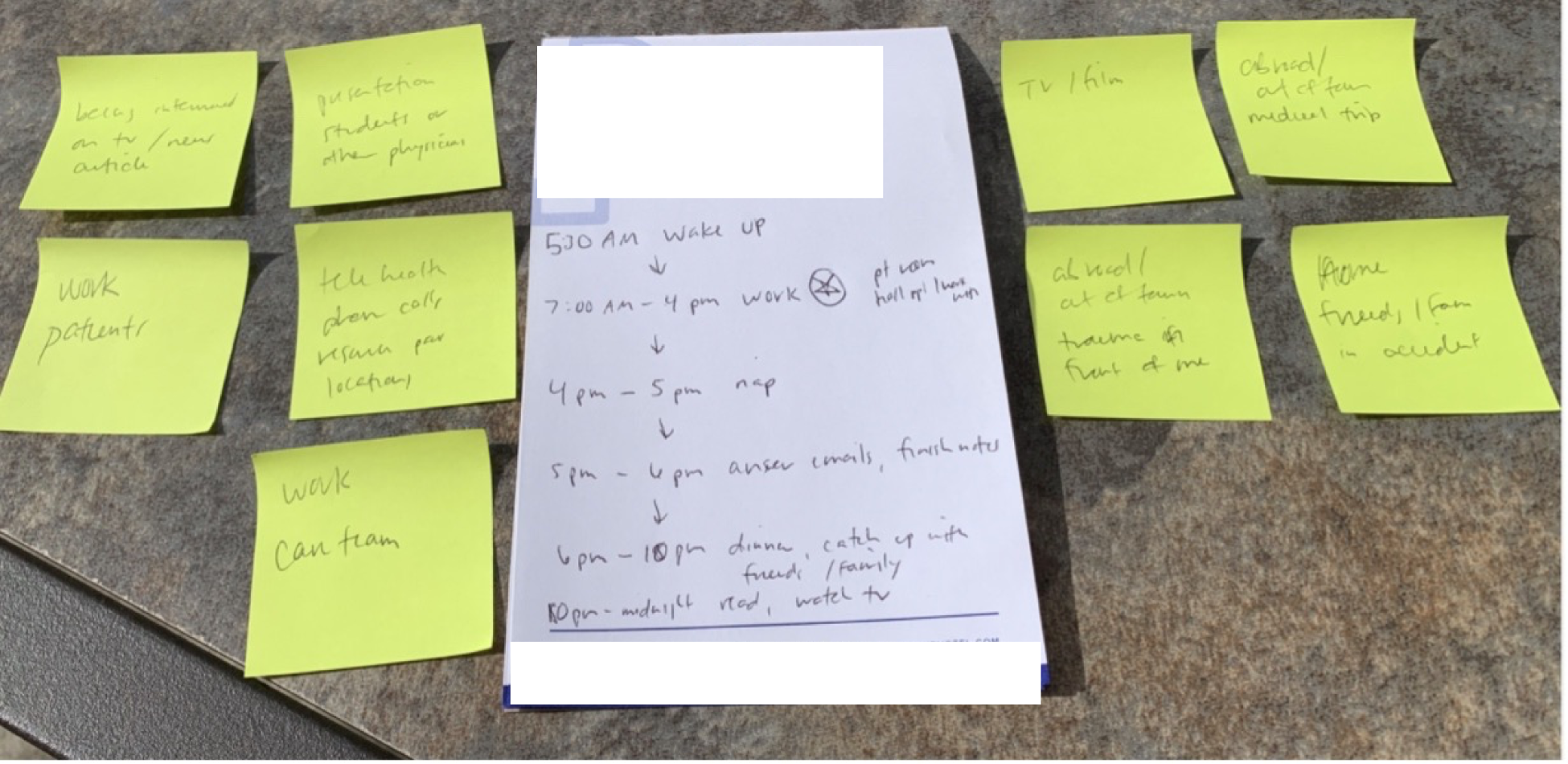

Supplement: S2 Fig — Scenario and rapid fire activities. (TIF) [file pgph.0002154.s002.tif]
